# The role of ageing and oxidative stress in intervertebral disc degeneration

**DOI:** 10.3389/fmolb.2022.1052878

**Published:** 2022-11-07

**Authors:** Pengfei Wen, Bolong Zheng, Binfei Zhang, Tao Ma, Linjie Hao, Yumin Zhang

**Affiliations:** ^1^ Department of Joint Surgery, Honghui Hospital, Xi’an Jiaotong University, Xi’an, Shaanxi, China; ^2^ Department of Spine Surgery, Honghui Hospital, Xi’an Jiaotong University, Xi’an, Shaanxi, China

**Keywords:** ageing, oxidative stress, intervertebral disc degeneration, intervertebral disc, reactive oxygen species

## Abstract

Intervertebral disc degeneration (IDD) is the primary cause of intervertebral disc (IVD) disease. With the increased ageing of society, an increasing number of patients are plagued by intervertebral disc disease. Ageing not only accelerates the decreased vitality and functional loss of intervertebral disc cells but also increases intracellular oxidative stress. Moreover, the speed of intervertebral disc ageing is also linked to high levels of reactive oxygen species (ROS) production. Not only is the production of ROS increased in ageing intervertebral disc cells, but antioxidant levels in degenerative intervertebral discs also decrease. In addition to the intervertebral disc, the structural components of the intervertebral disc matrix are vulnerable to oxidative damage. After chronic mitochondrial dysfunction, ROS can be produced in large quantities, while autophagy can eliminate these impaired mitochondria to reduce the production of ROS. Oxidative stress has a marked impact on the occurrence of IDD. In the future, IDD treatment is aiming to improve oxidative stress by regulating the redox balance in intervertebral disc cells. In summary, ageing and oxidative stress promote the degeneration of IVD, but further basic and clinical trials are needed to determine how to treat oxidative stress. At present, although there are many in-depth studies on the relationship between oxidative stress and degeneration of intervertebral disc cells, the specific mechanism has not been elucidated. In this paper, the main causes of intervertebral disc diseases are studied and summarized, and the impact of oxidative stress on intervertebral disc degeneration is studied.

## Introduction

With the process of increased societal ageing, the number of patients with intervertebral disc disease is increasing worldwide, resulting in a huge social and economic burden (Vos et al., 2012; Knezevic et al., 2021) A large number of studies have shown that Intervertebral disc degeneration (IDD) is the main cause of intervertebral disc disease and that IDD is associated with the severity of the disease (Takatalo et al., 2011). The degenerative process of IDD includes structural damage to the intervertebral disc and changes in various components. The intervertebral disc (IVD) is a fibrocartilaginous structure that not only provides flexibility between the vertebral bodies but also transmits compressive loads. It is very different from the connective tissue in the body; for example, degenerative ageing occurs early in life (Boos et al., 2002). Histological studies revealed reduced blood supply to the vertebral body starting in the second decade of life, and according to current research reports, there is a positive correlation between IDD prevalence and increasing age (Benneker et al., 2005). Relevant studies have shown that the cells in the IVD are closely related to changes in the nutrition of the matrix components, and their survival and nutrition depend on the nutrition in the matrix. The ageing of the IVD has dual hazards: 1. It leads to the loss of intervertebral cell viability and function; and 2. It releases substances such as matrix proteases and chemokines (Khan et al., 2017). The nonvascular characteristics of IVD affect the clearance of ageing IVD cells, inducing inflammation and catabolism, which affect the IVD microenvironment and accelerate IDD (Che et al., 2020). Therefore, understanding the interaction between spinal tissues during degeneration will enhance our understanding of the pathogenesis of this condition and will help to develop new and more effective treatments for IDD.

Oxidative stress (OS) refers to a state of imbalance between oxidation and antioxidant effects in the body. Loss of redox balance can lead to damage to key biological molecules and cells, potentially affecting organisms as a whole (Ďuračková, 2010). According to the latest research, the occurrence of IDD is closely related to OS caused by ROS (Hou et al., 2014; Dimozi et al., 2015). For example, a series of changes that affect gene expression and metabolism and the speed of IVD senescence have also been linked to the levels of ROS production (Vo et al., 2013). Therefore, we discuss the significance of IVD cell senescence in the pathogenesis of IDD and the significance of OS in IDD Elucidating the effects of ageing and OS on IDD will aid in elucidating the pathogenesis of IDD and developing a promising treatment.

## Mechanism of intervertebral disc degeneration

**FIGURE 1:**
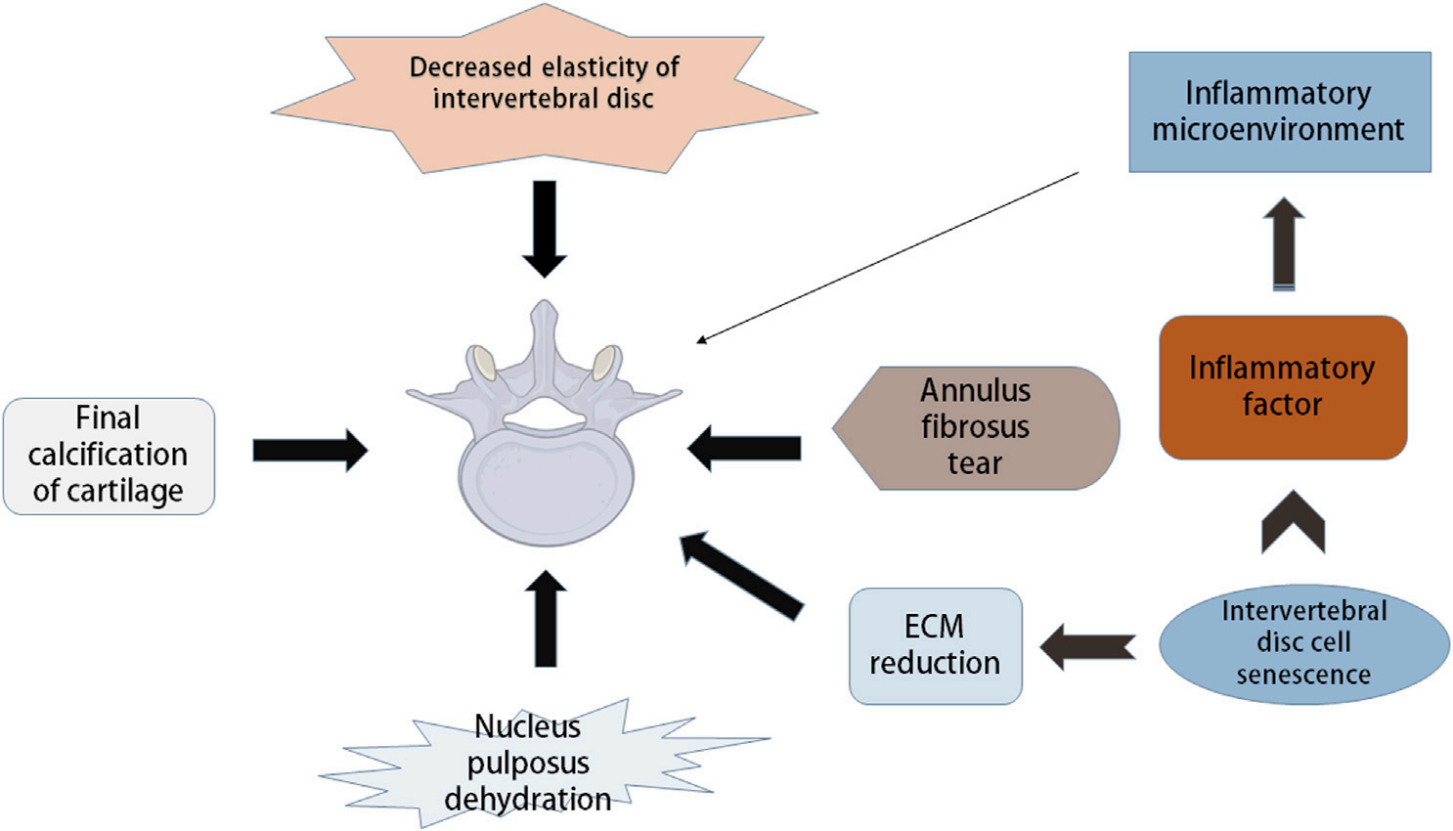
Pathological manifestation and mechanism of intervertebral disc degeneration. Intervertebral disc degeneration (IDD) includes many aspects, such as decreased disc elasticity, cartilage end-plate calcification, reduced moisture in nucleus pulposus, annulus fibrosus tears, and changes in extracellular matrix (ECM) components. Intervertebral disc cell senescence plays a major role in IDD, which can cause the reduction of ECM, and can secrete inflammatory factors leading to the inflammatory state of intervertebral disc microenvironment, thus causing IDD.

The IVD consists of three layers: annulus fibrosus, central nucleus pulposus and endplate (Colombini et al., 2008). The IVD is a vascular-free structure composed of fibrous tissue and cartilage (Boxberger et al., 2009). Cells and matrix are the basis of the normal function of IVD. IDD refers to the functional and structural failure of the IVD, which is related to its cellular pathogenesis and extracellular matrix (ECM) modification, including structural damage to the IVD and changes in cell number and other components, such as decreased elasticity of the IVD and tearing of the fibrous annulus (AF), leading to loss of water in the nucleus pulposus (NP) and calcification of the cartilage endplate (Figure 1). With increasing age, the composition of the ECM of the IVD changes significantly (Roughley, 2004). A variety of interdependent factors, including changes in mechanical load, reduced nutrition supply and genetic factors, are related to the progression of degradation cascades. Changes in ECM components of the IVD with age can be attributed to changes in function and the increase in death of cells that make up the IVD (Stokes and Iatridis, 2004; Battié and Videman, 2006; Zhao et al., 2007).

Although some mechanical stimulation is necessary for nutrient diffusion and matrix synthesis, excessive mechanical stimulation can lead to tissue loss and further change the strain distribution of the ECM between the vertebrae (Stokes and Iatridis, 2004). The discovery of ageing IVD cells has become the discovery point for further study of the pathogenesis of IDD. On the one hand, ageing IVD cells cannot produce new IVD cells, and the number of functional cells in the disc is reduced due to cell death. On the other hand, catabolic and proinflammatory phenotypes are the primary characteristic phenotypes of the senescence-related secretory phenotype (SASP) of disc cells (Le Maitre et al., 2007a). Ageing IVD cells may change their secretory pattern, thus altering the microenvironment of the IVD (van Deursen, 2014). They reduce the production of ECM *in vitro* while enhancing its degradation. In addition, the senescence of adjacent intervertebral cells and infiltration of immune cells that enhance inflammation of the degenerative disc microenvironment are due to proinflammatory cytokines secreted by senescent disc cells (Muñoz-Espín and Serrano, 2014).

In conclusion, phenotypic changes in ageing IVD cells, IVD ECM anabolism and catabolism are influencing factors in the IDD process.

## Oxidative stress and senescence

**FIGURE 2:**
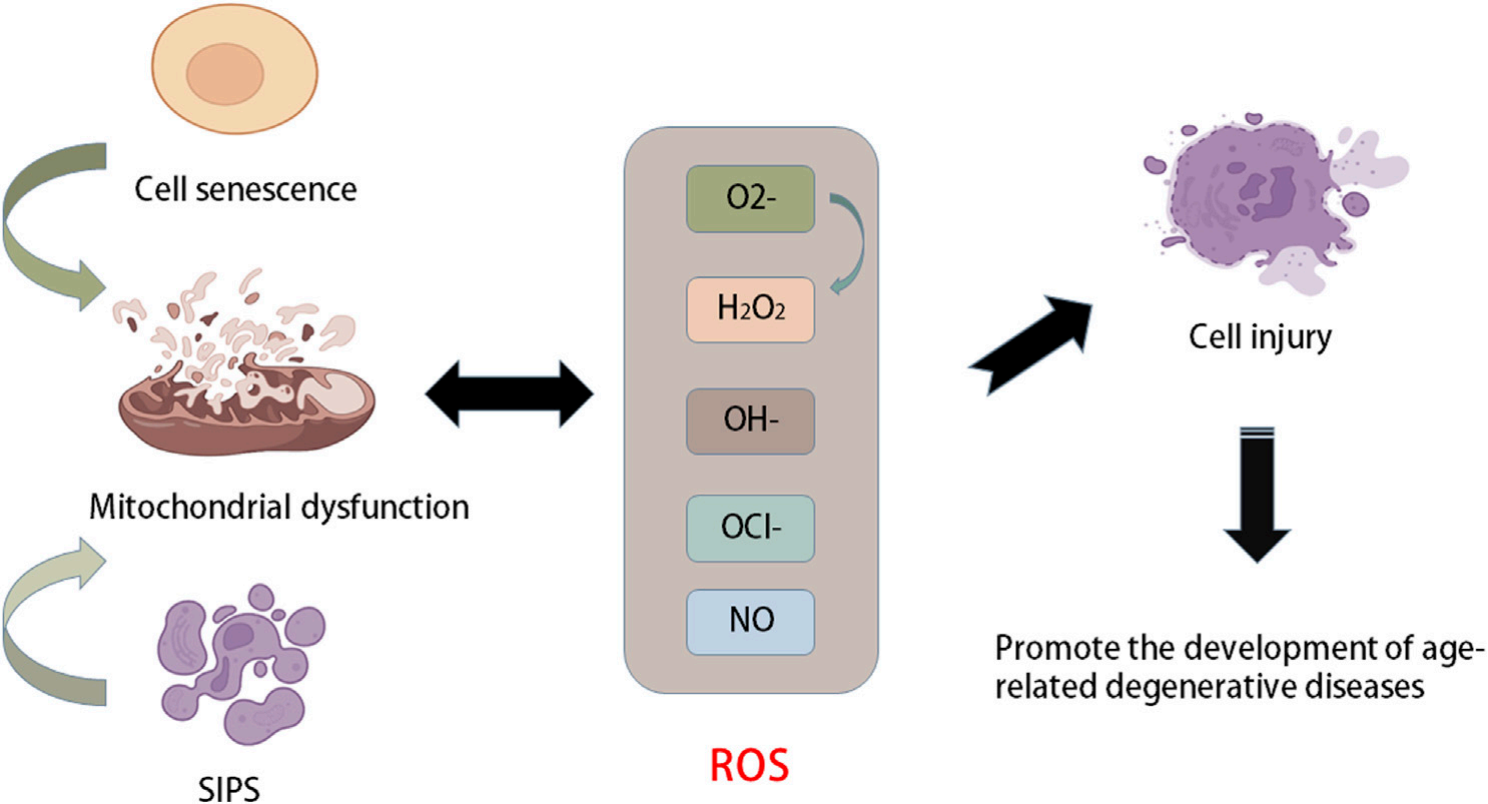
Relationship between aging and oxidative stress. Stress-induced premature senescence (SIPS) and natural senescence of cells can cause mitochondrial dysfunction, resulting in the production of a large number of ROS, such as oxygen anion (O_2_−), hydroxyl radical (OH−), hydrogen peroxide (H_2_O_2_), NO and hypochlorite (OCl−). When the production of ROS is greater than its own scavenging capacity, it will cause oxidative stress and cause cell damage and accelerate cell senescence, which further promotes the development of age-related degenerative diseases.

The balance between ROS production and removal directly determines intracellular redox homeostasis. ROS are inevitable in the process of cellular aerobic metabolism. When encountering stress, ROS will accumulate in cells, causing oxidative damage to proteins, DNA, and lipids and cell damage. Therefore, it has long been considered that ROS are a class of toxic molecules (Kim and Yim, 2015). As a key part of the antioxidant enzyme system, superoxide dismutase (SOD) is common in animals, plants and microorganisms and is inseparable from the occurrence and development of many diseases (Figure 2). SOD has strong oxidability and can catalyse the conversion of oxygen anion (O_2_−) to hydrogen peroxide (H_2_O_2_) primarily because O_2_− is produced by the Nox activity of the SOD enzyme and converts to H_2_O_2_. Reactive nitrogen species (RNS) are also classified as ROS because they have similar functions to ROS (Gorrini et al., 2013; Filomeni et al., 2015). Mitochondria are the primary production sites of ROS. During the process of electron transport, a small number of electrons leak and reduce O_2_ to O_2_− instead of H_2_O (Gille and Nohl, 2001). In addition, the main factors affecting intracellular redox homeostasis are the balance between ROS production and scavenging of ROS by nonenzymatic and enzymatic antioxidants, including reduced glutathione (GSH), catalase (CAT), glutathione peroxidase, and ascorbic acid (vitamin C) (Jones, 2008; Trachootham et al., 2008). These moieties are distributed in different parts of the cell and can interact with other molecules, including hydrooxidase and tocopherol, to remove ROS. Cellular senescence is an important mechanism that limits the proliferation of potential cancer cells (van Deursen, 2014).

Ageing is a complex process that leads to the functional decline of multiple tissues and organs. The ageing process is closely related to a variety of characteristics at the molecular, cellular and physiological levels, such as changes in the genome and epigenome, loss of protein homeostasis, decline in overall cellular and subcellular functions, and disorder of the signalling system. Another type of cellular senescence, stress-induced premature senescence (SIPS), is a result of the accumulation of genomic and mitochondrial DNA damage (Campisi, 2005; Acosta et al., 2008). In addition, SIPS cells are also related to senescence-related secretory phenotypes and can secrete a large number of inflammatory cytokines and matrix proteases, which decompose neighbouring cells and ECM to promote cellular degeneration (Rodier et al., 2009; Baker et al., 2011). Mitochondrial function decreases with age in various tissues and cell types (Ugalde et al., 2010). Mitochondria are the main source of ROS. Mitochondrial dysfunction increases the production of ROS and causes OS, which leads to cell damage and promotes the progression of ageing. Mitochondrial disease or dysfunction is ultimately an energy production problem because mitochondria are an important energy supply site in cells. Once mitochondria fail, it becomes more common to experience nutrient shortages (Harman, 1956; Jones, 2015). In addition, ageing and chronic degenerative diseases are accompanied by symptoms of mitochondrial dysfunction and inflammation. The former is one cause of ageing. When ageing occurs, mitochondrial DNA suffers oxidative damage to varying degrees, leading to damage to cellular energy metabolism, cell dysfunction, and even death. The relationship between the antioxidant capacity of mitochondria and changing age, as well as the increase in OS, cause physiological cell signal disorder, which damages cellular integrity and accelerates ageing.

## Effects of oxidative stress on intervertebral disc cells

**FIGURE 3:**
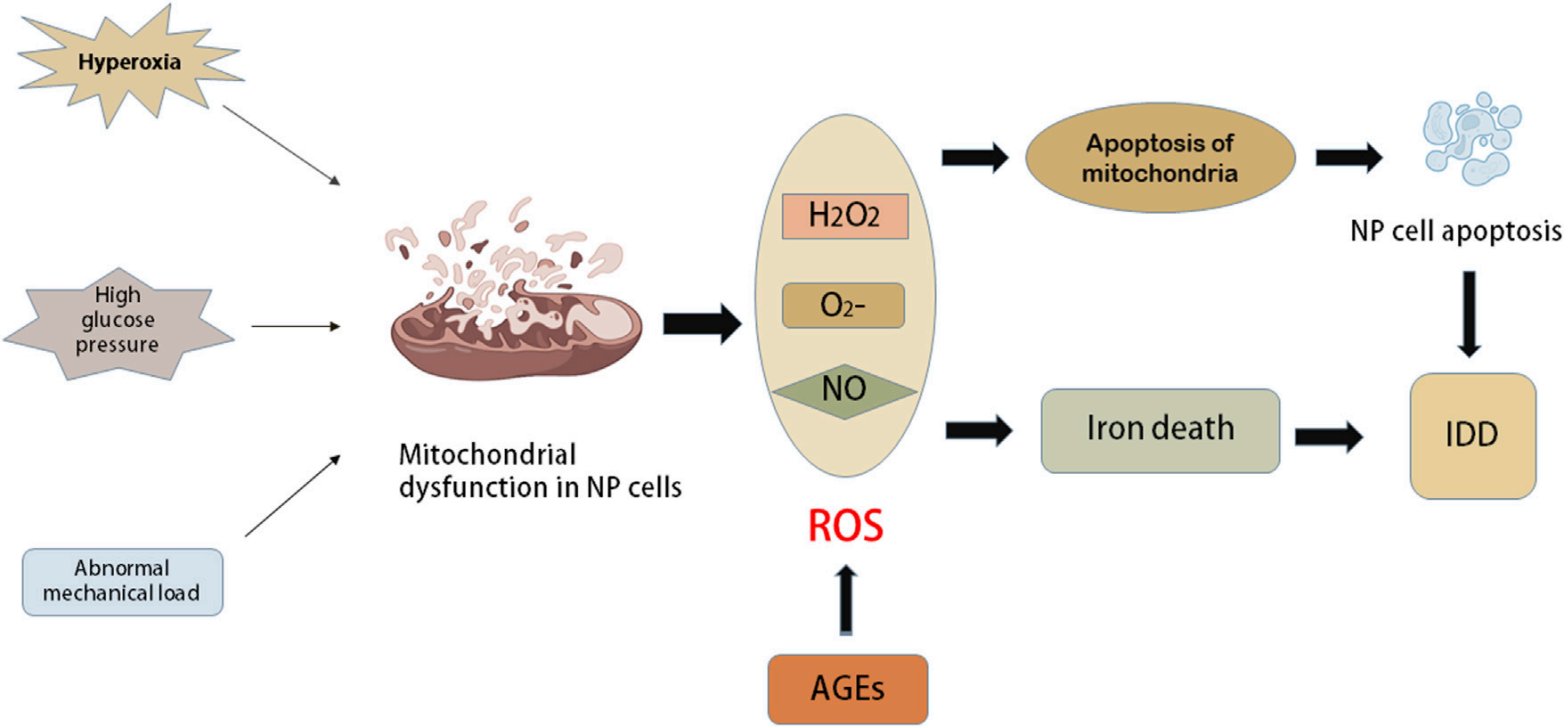
The role of oxidative stress in intervertebral disc cells. Mitochondria are the main source of ROS. Some harmful external factors such as hyperoxia, high glucose pressure and abnormal mechanical load can cause mitochondrial dysfunction in nucleus pulposus (NP) cells. The dysfunction of mitochondria causes a series of elevated levels of ROS, such as oxygen anion (O_2_−), hydrogen peroxide (H_2_O_2_) and NO. Oxidative damage of mitochondrial DNA and respiratory enzymes further lead to mitochondrial apoptosis and further accelerate mitochondrial dysfunction, which leads to a vicious circle between mitochondria and oxidative stress. Apoptosis of NP cells induced by mitochondrial apoptosis or functional impairment will accelerate the progress of IDD. The oxidative stress caused by the increase of ROS level can induce iron death, while iron death can inhibit the progress of IDD.

Apoptosis is one mechanism of cell death. Apoptosis can reduce the function and the number of surviving IVD cells, which is also the primary factor that induces IDD (Ding et al., 2013; Kepler et al., 2013). ROS are important to OS, and mitochondrial dysfunction is the main cause of excessive ROS. Previous studies have reported a decrease in mitochondria and an increase in mitochondrial respiration in human AF cells with IDD progression (Gruber et al., 2013). In addition, various stimuli, such as hyperoxic tension, high glucose pressure and abnormal mechanical load, have been shown to cause mitochondrial dysfunction in animal IVD cells (Ma et al., 2013; Jiang et al., 2014; Cai et al., 2015). These stimuli promote the production of ROS in NP and AF cells, and apoptosis can be further triggered by mitochondrial apoptosis (Ding et al., 2012). On the other hand, mitochondria are the main targets of ROS. Oxidative damage to mitochondrial DNA and respiratory enzymes further leads to mitochondrial dysfunction, causing a vicious cycle of mitochondrial and OS (Gruber et al., 2011). In addition, according to relevant studies, iron death is mainly an iron-dependent regulatory cell death characterized by lipid peroxidation of the cell membrane. Iron death is a recently discovered type of programmed cell death that plays an important role in tumour biology and therapy. Yang et al. (2021) found that OS can induce iron death in rat NP cells, while inhibition of iron death can slow the process of IDD *in vivo* (Figure 3).

H_2_O_2_ is a member of the ROS family, and it plays a wide role in IVDs. H_2_O_2_ can increase the permeability of the lysosomal membrane (Kim et al., 2009). Excessive ROS induces apoptosis of NP cells through the mitochondrial apoptotic pathway. In addition, H_2_O_2_ promotes ROS production and DNA damage in human NP cells (Zhou et al., 2016). In addition, overproduction of ROS induced by hyperglycaemia accelerates the senescence of annulus fibrosus and notochord cells in rats through the p16-Rb pathway (Krupkova et al., 2016). In fact, previous studies have reported excessive ROS production in degenerative IVDs, including a significant increase in NO levels in rat degenerative IVDs (Hou et al., 2014). Peroxynitrite is an effective oxidant formed by the interaction of O_2_ and NO in the body, which will further lead to tyrosine nitrosation and is a sign of excessive ROS (Hogg et al., 1992).

Nitrotyrosine-positive cells in human NP tissue increase with the development of IDD (Poveda et al., 2009). In a previous study, Schleicher et al. (1997) showed that advanced glycosylation end products (AGEs) are products of the oxidative modification of glycosylated proteins, including carboxymethyl lysine (CML) and pentosyl glycosides. CML and pentose accumulate in the degenerative IVD. To a certain extent, the degree of CML enhancement and IDD in the IVD will also increase (Nerlich et al., 1997) In addition, the key influencing factors of OS and apoptosis of human NP cells are the dysfunction of Sirtuin3 (SIRT3) and the mitochondrial antioxidant network. It is reported that oxidative stress induces excessive mitochondrial autophagy and further leads to cell death of human and rat NP cells (Xu et al., 2019). Another study found that OS can lead to mitochondrial dysfunction and mitosis in NP cells, while short hairpin RNA depletion of PINK1 further weakens mitosis and aggravates the senescence of NP cells under OS, indicating that PINK1-mediated mitosis is very important in protecting oxidative stress-related mitochondrial damage and cell senescence (Wang et al., 2018). Further studies have shown that salidroside upregulated Parkin may eliminate damaged mitochondria and promote the survival of NP cells by activating mitosis *in vitro*. *In vivo* experiments, Zhang et al. (2018) found that salidroside can inhibit the apoptosis of NP cells and improve the progress of IDD. Another study showed that melatonin alleviates intervertebral disc degeneration through mitochondrial autophagy induction and apoptosis inhibition (Chen et al., 2019). In addition, Urolitin A can induce mitochondrial autophagy and inhibit apoptosis and reduce intervertebral disc degeneration through activation of 5′-monophosphate-activated protein kinase (AMPK) signal pathway (Lin et al., 2020).

## The role of oxidative stress in the extracellular matrix

**FIGURE 4:**
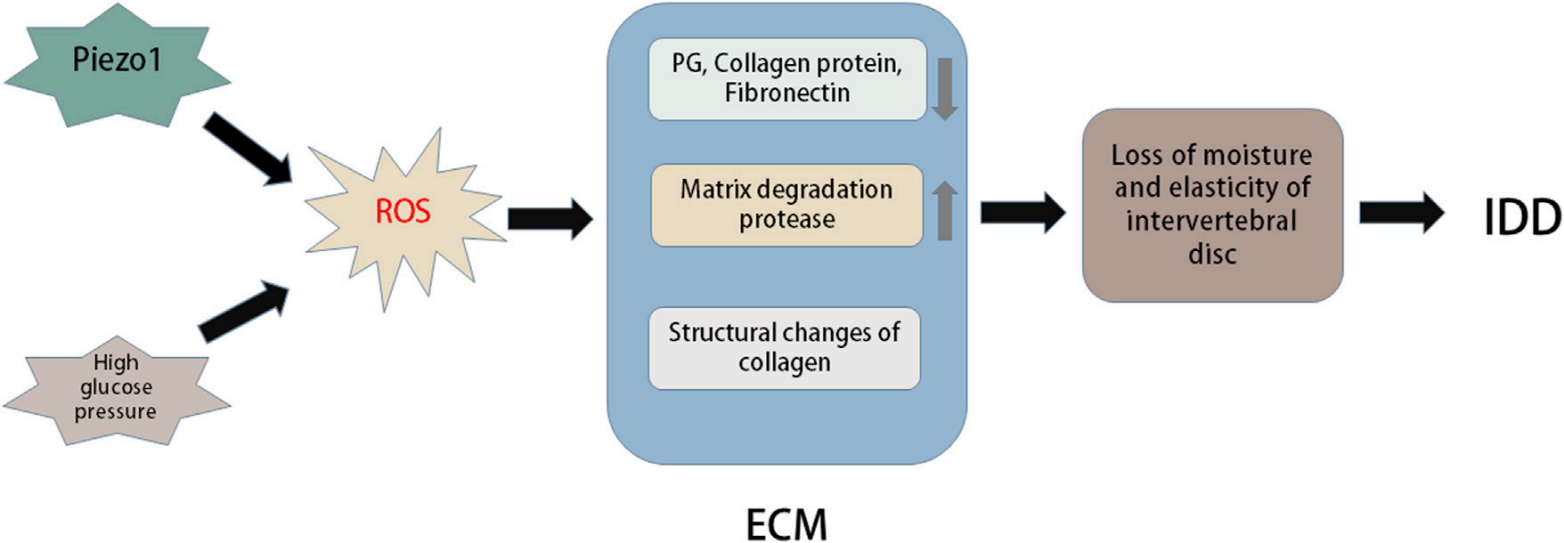
Oxidative stress in extracellular matrix of intervertebral disc. Piezo1 and high glucose environment can promote oxidative stress to induce more ROS production. Excessive ROS can cause changes in the structure and composition of extracellular matrix (ECM). For example, ROS can lead to a large loss of PG, collagen and fibronectin in intervertebral discs, oxidative damage of collagen I and collagen II lose their natural primary and secondary structures, and excessive ROS can cause the expression of matrix degradation proteases. These changes in ECM can cause reduced moisture and elasticity in the intervertebral disc, thus accelerating the progression of intervertebral disc degeneration (IDD).

The degradation of IVD mainly involves the reduction of water content and ECM decomposition. It represents the loss of steady-state metabolism, which is primarily caused by an imbalance between anabolism and catabolism, which also demonstrates that anabolism and catabolism play important roles in the IDD process (Horner and Urban, 2001; Sobajima et al., 2005). Moreover, metabolism of the ECM is closely related to the redox state in the IVD (Yang D. et al., 2014). The ECM of IVDs primarily includes the matrix network and collagen (Hwang et al., 2014). This network is essential for the IVD as a shock absorber to resist the mechanical load applied on the spine (Le Maitre et al., 2007b). Posttranslational oxidative modification of the IVD matrix occurs during IDD. Numerous studies have shown that H_2_O_2_ can significantly downregulate the expression of type II collagen and proteoglycans in human and rat IVD cells (Wei et al., 2008). Excessive production of ROS induced by anti-inflammatory cytokines or hyperoxia significantly inhibits matrix synthesis and regulates the expression of matrix-degrading proteases in human and rat IVD cells (Scharf et al., 2013) (Figure 4).

Some studies have found that AGEs increase IVD denaturation mainly by promoting cell apoptosis and preventing ECM metabolism. The primary components are pentoside and carboxymethyl lysine (Vistoli et al., 2013). Pentoside is distributed in collagen, and collagen molecules are cross-linked. Collagen stiffness and cartilage biomechanical dysfunction may play an important role with age (DeGroot et al., 1999). The subsequent significant loss of elasticity and stiffness of the IVD damages the mechanical function of the IVD and leads to IDD. The key influencing factor of ageing and apoptosis in human IDD is matrix hardness-activated Piezo1 (Wang et al., 2021). OS induced by high glucose may mediate apoptosis and imbalance of ECM metabolism through p38-MAPK activation in rat NP cells (Cheng et al., 2016).

## Oxidative stress and inflammation in intervertebral disc degeneration

Inflammation can maintain physiological and pathological homeostasis during infection, but excessive proinflammatory factors can cause damage to the body, with persistent and intense inflammation leading to serious diseases (Risbud and Shapiro, 2014; Žerovnik et al., 2020). Park et al. (2007) found that compared to the control group, patients with lumbar disc herniation exhibited higher levels of serum IL-2, IL-6, IL-8, and tumour necrosis factor. In a pathological model of IDD, it was found that NP cells and fibrocartilage rings (fibrocartilage tissue containing NP) were abnormally regulated by proinflammatory molecules released by macrophages, T cells and neutrophils, indicating that IVD-derived cells can initiate or amplify inflammation (Kraychete et al., 2010). Based on these findings, inflammation plays an important role in the process of IDD. Moreover, chemokines secreted by IVD cells reduce cell vitality and cause functional degradation, resulting in a vicious cycle (Risbud and Shapiro, 2014).

In general, inflammatory tissue is associated with increased levels of active substances (ROS and RNS) produced by immune cells and is essential to help fight foreign pathogens (Lei et al., 2015). These active substances react with various metabolic substances in biological molecules. Nitrosation substances affect cellular signal transduction and regulate the inflammatory response (Chiurchiù and Maccarrone, 2011). Once the cellular pro-oxidation and antioxidation system fails, excessive inflammation will occur, and the production of ROS will increase (Miki and Funato, 2012). Degeneration of IVD is related to local increases in IL-1 β (Le Maitre et al., 2005), which may be because IL-1 β can induce apoptosis through mitochondrial dysfunction and endoplasmic reticulum stress (López-Armada et al., 2006; Caramés et al., 2008; Kim et al., 2010). Elevated ROS activates multiple signalling pathways of intervertebral cells during oxidative stress, including the NF-κB and mitogen-activated protein kinase (MAPK) pathways (Feng et al., 2017). NF-κB is a transcription factor that regulates the gene encoding proinflammatory cytokines and is also a potential therapeutic target for various inflammatory diseases (Wang et al., 2009).

ROS can induce or mediate activation of the MAPK pathway (McCubrey et al., 2006). The metabolism of NF-κB in NP cells is regulated by H_2_O_2_ and peroxynitrite (Loukili et al., 2010; Risbud and Shapiro, 2014). Inflammatory factors of receptor heat protein domain related protein 3 (NLRP3), which exert adverse effects on metabolism, aggravate the apoptosis of NP cells and lead to the degeneration of IVD (Tang et al., 2018).

In addition, mitochondrial dysfunction and the production of mitochondrial ROS promote the activation of NLRP3 inflammatory bodies. Mitochondria provide cellular energy and can also regulate cellular functions, such as calcium concentration in the internal cellular environment, signal transduction, and apoptosis. Loss of mitochondrial function and impaired ATP production were found to induce cell death. In the past decade, increasing evidence has demonstrated that mitochondria are involved in NLRP3 inflammasome activation and pyroptosis, playing an important role in this process. Mitochondria participate in the regulation of the natural immune system, which has been an important discovery in recent years (Tang et al., 2018).

## Autophagy and oxidative stress in intervertebral disc degeneration

Autophagy provides energy by self-digesting damaged or ageing organelles to protect cells from various harmful external stimuli (Kim and Lee, 2014). ROS are produced in the mitochondria, and this process can regulate autophagy. High levels of ROS can be produced after chronic damage to mitochondrial function. Autophagy can eliminate these impaired mitochondria to reduce the production of ROS. Through this mechanism, autophagy can cut off the source of OS and reduce cellular damage (Filomeni et al., 2015).

Many studies have shown that the increase in intracellular H_2_O_2_ concentration not only enhances the AMPK and its mediated cellular adaptation but also maintains redox homeostasis to a certain extent (Sandström et al., 2006; Horie et al., 2008; Irrcher et al., 2009). For example, excessive ROS activate the MAPK pathway in plants and animals to induce autophagy, and a large number of studies have experimentally confirmed this (Jalmi and Sinha, 2015; Liu et al., 2020). When cells are exposed to ROS/NOS produced by metabolism or stress, nuclear factor (erythroid-derived-2)-like 2 (Nrf2) can maintain the dynamic balance of cells. A recent study demonstrated that Nrf2 is involved in fine-tuning the autophagic process in response to OS levels and functions in a feedback loop, binding to AMPK, which is essential for the induction of autophagy (Kapuy et al., 2018).

**FIGURE 5:**
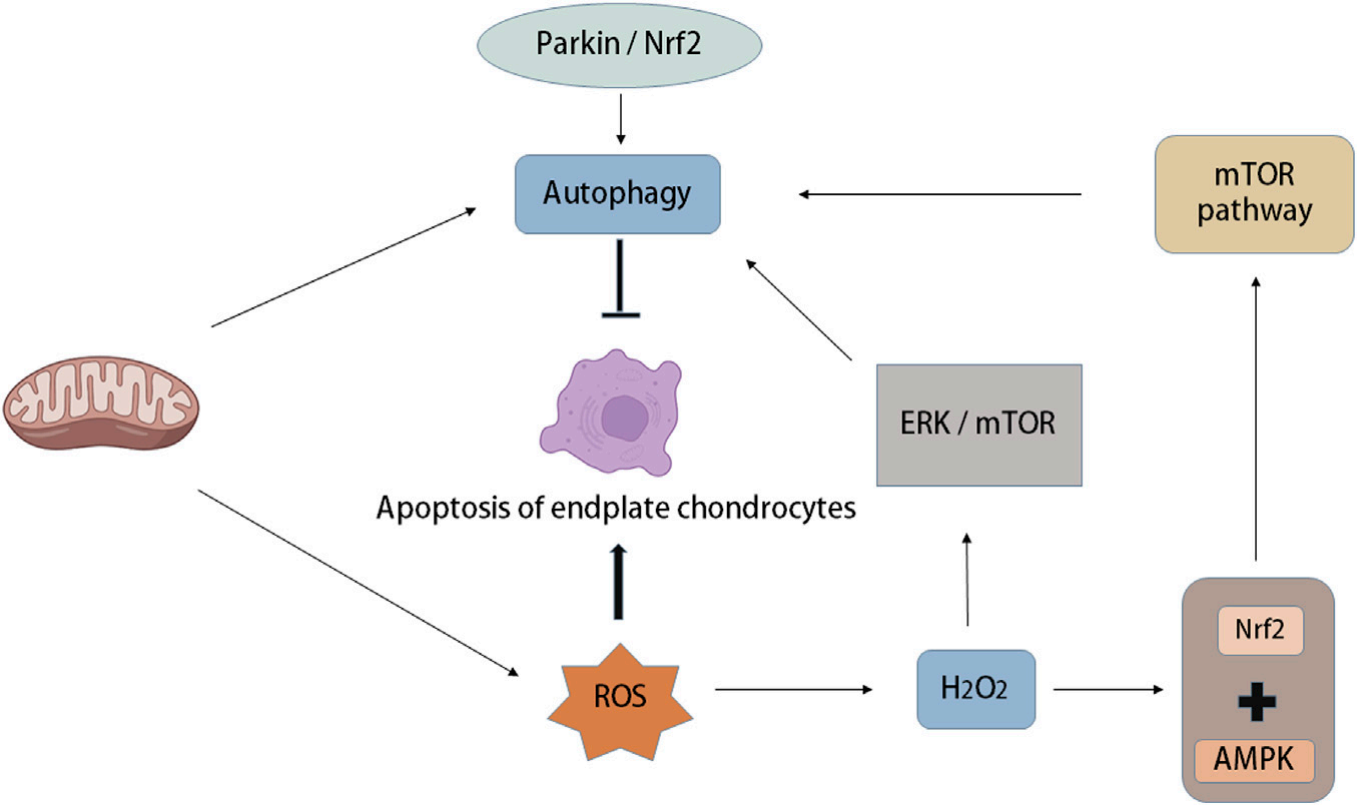
Autophagy and oxidative stress in intervertebral disc degeneration. Mitochondria are not only the place where ROS is produced, but also the organelles that regulate autophagy. Oxidative stress can increase autophagy and apoptosis of intervertebral disc endplate chondrocytes, while the increase of autophagy activity can prevent endplate chondrocyte apoptosis, in which oxidative stress-induced autophagy of endplate chondrocytes is mTOR-dependent. A large amount of ROS was produced after mitochondrial injury, in which the increase of H_2_O_2_ concentration led to the activation of 5′-adenosine monophosphate-activated protein kinase (AMPK), while the combination of Nuclear factor (erythroid-derived-2)-like 2 (Nrf2) and AMPK induced autophagy through the down-regulation mechanism of mTOR. In addition, Parkin and Nrf2 can prevent apoptosis of intervertebral endplate chondrocytes induced by oxidative stress by inducing mitochondrial autophagy.

OS increases autophagy and apoptosis of IVD chondrocytes, but with the increase in autophagy *in vivo*, cartilage apoptosis is prevented, in which OS-induced autophagy of endplate chondrocytes is mTOR-dependent (Chen et al., 2017). There are many relevant studies, and autophagy occurs not only in rats but also in human degenerative IVD cells (Ye et al., 2011; Gruber et al., 2015). Research has demonstrated that autophagy occurs in degenerative IVDs in rats and humans, in which ROS are key regulators of autophagy *in vitro*. For example, H_2_O_2_ promotes autophagy in rat NP cells through the ERK/mTOR signalling pathway, and autophagy inhibition significantly reduces the incidence of apoptosis induced by H_2_O_2_. In addition, H_2_O_2_ leads to OS and apoptosis of chondrocytes, while Parkin and Nrf2 prevent apoptosis of intervertebral endplate chondrocytes induced by OS by inducing mitochondrial autophagy and antioxidant defence (Kang et al., 2020) (Figure 5).

IVD compression can accelerate IDD (Desmoulin et al., 2020), and autophagy can be activated by ROS to protect the IVD and delay IDD (Ma et al., 2013). Moreover, OS induced by high glucose levels can also promote mitochondrial damage, leading to autophagy in rat cells (Park and Park, 2013). Tang et al. (2019) showed that excessive OS leads to the upregulation of autophagy, and autophagy acts as an antioxidant response in IDD.

## Delaying intervertebral disc degeneration by reducing oxidative stress

OS has a marked impact on the IDD process. It not only regulates the vitality and function of IVD cells but also play an important role in the ECM structure of IVDs, representing a promising treatment to improve the IDD process by restoring the redox balance of IVD cells.

Studies have found that AGEs accumulate in the IVDs of diabetic mice or rats, which accelerates disc degeneration (Illien-Junger et al., 2013). However, oral pyridoxamine (an AGE inhibitor) can reduce the production and inflammation of ROS in the IVD of diabetic mice, thus slowing the progression of IDD (Fields et al., 2015). GSH is the main antioxidant in living cells. Moreover, antioxidants such as N-acetylcysteine (NAC) eliminate the catabolism of excess ROS and TNF-α *in vitro* (Suzuki et al., 2015). Oral administration of NAC has been shown to prevent IDD in rat degenerative models. These findings provide new ideas and new targets for continuing to perform research on relevant new mechanisms and precise prevention and treatment based on IDD pathogenesis (Suzuki et al., 2015).

In addition, Lu et al. (2021) found that under OS conditions, NP cell depletion and the pathogenesis of IVD are related to ferroportin (FPN) dysfunction, while ferritin-dependent iron homeostasis has a protective effect on IDD induced by OS. Che et al. (2020) found that p16 plays an important role in the pathogenesis of IVDD, and its deletion weakens IVD by promoting cell cycle and inhibiting SASP, cell senescence and oxidative stress. Xia et al. (2019) found that exocrine bodies derived from mesenchymal stem cells can improve IDD through antioxidant and anti-inflammatory effects.

Some active oxygen scavengers, such as pyrroloquinoline quinone (PQQ) (Zhang et al., 2013), fullerene (Anilkumar et al., 2011) and fullerol (Yang D. et al., 2014), can effectively improve OS induced by excessive ROS. PQQ is not only the primary cofactor of mitochondrial dehydrogenase but also a scavenger of ROS, playing an important role in the production of ROS in mitochondria (Zhang et al., 2013). PQQ performs an antioxidant function and can prevent some OS reactions in mitochondria (Xiong et al., 2011).

In IVD cells, PQQ inhibits the excessive production of ROS induced by H_2_O_2_ can prevent the apoptosis of rat NP cells induced by H_2_O_2_ in rat NP cells, and then protected rat NP cells from H_2_O_2_ induced apoptosis *in vitro* (Yang et al., 2015). Fullerenes can protect rats from lipid peroxidation in tissues (Vapa et al., 2012). Fullerol, a derivative of fullerene, can reduce the production of ROS in human NP cells and slow IDD by promoting matrix synthesis and inhibiting heterotopic ossification (Yang D. et al., 2014).

Polyphenol is a secondary metabolite produced by plants and has a strong antioxidant capacity, which mainly depends on the ability to express antioxidant genes such as catalase and inhibit the expression of oxidation-promoting genes (Akhtar and Haqqi, 2012). Resveratrol (RSV) is a polyphenol compound that exists in various plants. It has been widely used in many settings (Jiang et al., 2014; Wang et al., 2016). For example, mitochondria are important targets for RSV, and RSV can regulate ROS biosynthesis in mitochondria and regulate energy metabolism through interactions with other genes (Bastin et al., 2011; Zhou et al., 2014).

GSH can inhibit the apoptosis and matrix decomposition of human NP cells induced by H_2_O_2_ and reduce the production of ROS in human NP cells (Yang X. et al., 2014). As a precursor of GSH, oral N-acetylcysteine can inhibit OS, matrix catabolism and inflammation of IVDs in rats, delaying the development of IDD (Dimozi et al., 2015). Oestrogen is a steroid hormone primarily produced in the ovary that has been shown to have significant regulatory effects on a variety of human systems (Cauley, 2015). It can also maintain and promote the metabolism of bone matrix, promote calcium and phosphorus salts to undertake in fractures, and maintain normal fracture oestrogen and parathyroid hormone to maintain a balance. With increasing age, if oestrogen levels are low, a series of diseases, such as arteriosclerosis and osteoporosis, can occur (Cauley, 2015). Oestrogen can also regulate IVD metabolism and affect antioxidation (Jin et al., 2018). Insufficient oestrogen can also cause IVD disease and calcification, which significantly aggravate IDD (de Quadros et al., 2019). Oestrogen supplementation can repair these pathological changes and endplate calcification of IVD, promoting the expression of ECM and restoring the hydrophilicity of IVD (Liu et al., 2019).

Several growth factors can protect IVD cells from harm. Bone morphogenetic protein 7 can inhibit the apoptosis-promoting effect of H_2_O_2_ on human NP cells *in vitro* and helps human NP cells maintain matrix synthesis under OS (Wei et al., 2008). Insulin-like growth factor-1 improves H_2_O_2_ -induced premature ageing of human atrial fibrillation cells *in vitro* (Gruber et al., 2008).

## Future prospects and conclusion

The pathological changes of IVD are important to IDD Exploring the effect of OS on IDD provides important research ideas for our future research on the pathogenesis of IDD. With the progress of science and technology, an increasing number of researchers continue to deepen the understanding of the pathogenesis of IDD, although its pathogenesis is not fully understood at present. *In vivo* and *in vitro* experiments have made it clear that OS is important to the pathogenesis of IDD, which opens a new way to understand IDD. OS has a great impact on the IDD process. It not only regulates the vitality and function of IVD cells but also plays an important role in the ECM structure of IVDs. Targeting OS is a promising treatment to improve the IDD process through regaining redox balance in IVD cells. Antioxidant therapy is also considered a new and promising treatment for IDD, even though there is not sufficient evidence *in vivo* that antioxidants delay the establishment of IDD. In short, ageing and OS promote the degeneration of IVD, but additional basic research and clinical trials are needed to determine how to treat OS.
